# New features on the survival of human-infective *Trypanosoma rangeli* in a murine model: Parasite accumulation is observed in lymphoid organs

**DOI:** 10.1371/journal.pntd.0009015

**Published:** 2020-12-28

**Authors:** Luciana de Lima Ferreira, Fernanda Fortes de Araújo, Patricia Massara Martinelli, Andrea Teixeira-Carvalho, Juliana Alves-Silva, Alessandra Aparecida Guarneri

**Affiliations:** 1 Vector Behavior and Pathogen Interaction Group, Instituto René Rachou, Fundação Oswaldo Cruz-FIOCRUZ, Belo Horizonte, Minas Gerais, Brazil; 2 Integrated Research Group in Biomarkers, Instituto René Rachou, Fundação Oswaldo Cruz-FIOCRUZ, Belo Horizonte, Minas Gerais, Brazil; 3 Departamento de Morfologia, Instituto de Ciências Biológicas, Universidade Federal de Minas Gerais, Belo Horizonte, Minas Gerais, Brazil; Universiteit Antwerpen, BELGIUM

## Abstract

*Trypanosoma rangeli* is a non-pathogenic protozoan parasite that infects mammals, including humans, in Chagas disease-endemic areas of South and Central America. The parasite is transmitted to a mammalian host when an infected triatomine injects metacyclic trypomastigotes into the host′s skin during a bloodmeal. Infected mammals behave as parasite reservoirs for several months and despite intensive research, some major aspects of *T*. *rangeli*-vertebrate interactions are still poorly understood. In particular, many questions still remain unanswered, e.g. parasite survival and development inside vertebrates, as no parasite multiplication sites have yet been identified. The present study used an insect bite transmission strategy to investigate whether the vector inoculation spot in the skin behave as a parasite-replication site. Histological data from the skin identified extracellular parasites in the dermis and hypodermis of infected mice in the first 24 hours post-infection, as well as the presence of inflammatory infiltrates in a period of up to 7 days. However, qPCR analyses demonstrated that *T*. *rangeli* is eliminated from the skin after 7 days of infection despite being still consistently found on circulating blood and secondary lymphoid tissues for up to 30 days post-infection. Interestingly, significant numbers of parasites were found in the spleen and mesenteric lymph nodes of infected mice during different periods of infection and steady basal numbers of flagellates are maintained in the host′s bloodstream, which might behave as a transmission source to insect vectors. The presence of parasites in the spleen was confirmed by fluorescent photomicrography of free and cell-associated *T*. *rangeli* forms. Altogether our results suggest that this organ could possibly behave as a *T*. *rangeli* maintenance hotspot in vertebrates.

## Author summary

*Trypanosoma rangeli* development inside vertebrates is a highly controversial field of study, since there is no evidence of parasite multiplication in host tissues. One of the difficulties that turns it even more complex, it’s the challenge to maintain *T*. *rangeli* infectivity, since its maintenance in culture decreases parasite’s ability to infect its hosts. Our group developed a methodology in which parasites are kept in frequent passages through triatomines and mice, assuring its infectivity and allowing the development of studies more similar to natural transmission. In this study we used qPCR, flow cytometry and histological techniques to evaluate parasite persistence in different tissues and organs of mice infected by the bite of infected bugs, mimicking a natural infection. We found that after one week of infection, parasites as well as inflammatory infiltrates disappear from the inoculation site at the host´s skin. Evaluation of infected mice for a period of 30 days demonstrated that a stable basal amount of parasites was detected in circulating blood, although an increased amount of *T*. *rangeli* DNA was found in mesenteric lymph nodes and spleen. In spleen, through marking the parasite with a monoclonal antibody we confirmed the presence of live parasites which were seen in apparent close association with cells and also inside them as apparently amastigote forms, indicating that the parasite is preserved and possibly replicates in these tissues. Our study provides new insights into the interaction of *T*. *rangeli* with its vertebrate hosts.

## Introduction

*Trypanosoma rangeli* and *Trypanosoma cruzi* are the two species of trypanosomes that infect humans in the Americas [1]. Contrarily to *T*. *cruzi*, which is the causative agent of Chagas disease, *T*. *rangeli* infection has not been associated to symptomatic pathologies or diseases. Nevertheless, infections with *T*. *rangeli* trigger a humoral immune response in humans, which can produce cross-reactivity with *T*. *cruzi* antigens in different immunoserological tests, causing misdiagnosis and affecting epidemiological analyses [2–6]. Despite the fact that vector-parasite interactions between *T*. *rangeli* and triatomines have been extensively studied and characterized (reviewed by [7]) its development inside vertebrate hosts is still surrounded by much speculation, especially regarding parasite survival and replication. Artificially-infected mice usually present low frequencies of circulating blood forms of *T*. *rangeli*, even during infection peak, which has been described to occur between the 3^rd^ and 5^th^ days of infection [8–10]. Reports have shown that parasitemia decreases over time until trypanosome infection becomes unapparent [9,10]. *In vitro* assays using different cell lineages have failed to show amastigote or replicative forms and are marked by low infectivity, suggesting an absence of intracellular multiplication [8,11,12]. Nevertheless, *T*. *rangeli* transmission has been reported for triatomines feeding on long-term chronically infected mice showing up to 12 months of infection [13–15], suggesting that the parasite must undergo replication to establish a systemic infection in mammalian hosts. Additionally, sub-cutaneous or intradermal inoculation of parasite-cultured forms have been used as infection strategy in most studies aimed to find *T*. *rangeli* in vertebrates hosts [9,14,16,17]. Such approaches do not essentially reflect vector-born natural ways of transmission and possibly create an infection-establishment bias.

As solenophagic insects, triatomines take their meal directly from the lumen of peripheral blood vessels [18] in a process whereby saliva is released during the whole feeding event to aid vessel localization and maintenance of blood flow during ingestion [19]. Transmission of *T*. *rangeli* to vertebrate hosts occurs when infected triatomines feed on a mammal, inoculating saliva and metacyclic trypomastigotes into their skin [20]. In this sense, during the feeding process of *T*. *rangeli*-infected triatomines, it seems reasonable to expect that part of the parasites would be delivered directly into peripheral blood vessels, as well as some should be retained on the dermis. We have previously shown that a single *Rhodnius prolixus* 5^th^ instar nymph can release around 50,000 *T*. *rangeli* metacyclic trypomastigotes during blood uptake [15]. This impressive parasite number assures the establishment of the infection in ~90% of the mice exposed to infected nymphs. The resistance to complement system by metacyclic trypomastigotes might guarantee their survival during the first hours of infection [15].

Aiming to investigate possible replication sites of *T*. *rangeli* inside a murine host, we evaluated whether the parasite can colonize the host´s skin at the bite site or is targeted to other tissue/organs. In order to do this, we evaluated the persistence of *T*. *rangeli* at the bite site and quantified parasite load in the blood and certain lymphoid organs, at different times of a 30-day infection interval. Mice were infected by the bite of triatomines containing trypomastigote forms in salivary glands to simulate natural transmission. Our results showed, for the first time, an accumulation of *T*. *rangeli* DNA in spleen and lymph nodes of long-term infected mice. Parasite viability was confirmed by RNA amplification and imaging flow cytometry suggesting that these tissues can behave as trypanosome replication niches from which they are delivered to circulating peripheral blood.

## Methods

### Ethics statement

All experiments using live animals were performed in accordance with FIOCRUZ guidelines on animal experimentation and were approved by the Ethics Committee in Animal Experimentation (CEUA/FIOCRUZ) under the approved protocol number LW 61/12. The protocol is from CONCEA/MCT (http://www.cobea.org.br/), which is associated with the American Association for Animal Science (AAAS), the Federation of European Laboratory Animal Science Associations (FELASA), the International Council for Animal Science (ICLAS) and the Association for Assessment and Accreditation of Laboratory Animal Care International (AAALAC).

### Insect rearing

*Rhodnius prolixus* were obtained from a colony originally established with insects collected in Honduras around 1990 and has been maintained by the Vector Behavior and Pathogen Interaction Group. Insects were reared at 26±1°C, 65±10% RH under natural illumination cycle. Monthly feeding of the colony was performed on chicken or mice, which were previously anesthetized with intraperitoneal injections mixtures of ketamine (20 mg/kg; Cristália, Brazil) and detomidine (0.3 mg/kg; Syntec, Brazil) or ketamine (150 mg/kg; Cristália, Brazil) and xylazine (10 mg/kg; Bayer, Brazil), respectively.

### Parasite strain

*Trypanosoma rangeli* Choachi strain originally isolated from naturally infected *R*. *prolixus* from Colombia [3] was used to infect insect vectors. Parasites were cultured *in vitro* in liver-infusion tryptose (LIT) supplemented with 15% fetal bovine serum (FBS), 100 μg of streptomycin/ml and 100 units/ml of penicillin at 27°C, and passaged twice a week. Loss of parasite infectivity was prevented by continuous full cycle infections on triatomine/mouse hosts every three months [21,22].

### Mice

Male mice from *Swiss Webster* lineage, at the age of around 60 days, and weighing approximately 40 g were used as vertebrate host model in this study. Animals were obtained from in-house animal care facility and subjected to anesthetic procedures (described above) prior to experimentation.

### *Rhodnius prolixus* infection

Third instar nymphs were fed on an artificial feeding apparatus containing heat-inactivated (56°C/30 min) citrated rabbit blood with a suspension of *T*. *rangeli* culture epimastigotes (1x10^5^ parasites/ml) at 37°C. Citrated rabbit blood was obtained from CECAL (Centro de Criação de Animais de Laboratório), Fiocruz, RJ. Epimastigotes were retrieved from a 10-day culture, washed and resuspended in 50 μl of sterile phosphate buffered saline (PBS; 0.15M NaCl in 0.01M Na_3_PO_4_; pH 7.4), before being added to the blood. As the proportion of insects in which parasites cross the intestinal barrier to complete development in salivary glands is variable and normally do not exceeds 50% (even in those bugs collected in the wild, for a review see [7]), an intracoelomic inoculation was performed in the same nymphs seven days after they had reached the 4^th^ instar [23]. Briefly, nymphs were inoculated in the thoracic pleura with 1 μl of PBS containing 1x10^5^ culture epimastigotes/ml. Twenty four hours after the inoculation, the nymphs were allowed to feed *ad libitum* on anesthetized mice. Insects with at least a month of hemocoelomic infection were used in the experiments.

### Mice infections

Three different forms of infection were used in the present work to fulfill different requirements related to specific experiments. It has been previously showed that during the probing period the triatomine maxillae move and release the saliva in different points of the skin [19]. Therefore, to maximize the amount of parasites delivered at the site of inoculation and increase the chances of visualizing parasites in a specific region, we exposed a delimited skin area to large amounts of metacyclic trypomastigotes. For this purpose, salivary glands (SG) of five infected 5^th^ instar nymphs were dissected, washed in PBS and transferred to a 0.2 ml tube containing 20 μl of M199 culture medium (M3769; Sigma-Aldrich, St. Louis, MO, USA). SG were then disrupted and had their contents homogenized in order to obtain a mixture of free metacyclic trypomastigotes. Infected nymphs with more than 30 days post-inoculation date were used to guarantee a proportion of metacyclic trypomastigotes over 95% in the SG [24]. Mice were anesthetized and had the hairs removed from an area of ~5 mm^2^ diameter in the central region of the abdomen. To allow the entry of parasites in the skin, this area was superficially punctured with a needle (0.30x13 mm; BD Precision GLIDE, Brazil). In total, 10 punctures were performed with a distance of ~1 mm from each other. Immediately after the puncture, the parasite suspension was applied to the punctured area. Mice were monitored for 10 min in order to prevent movements, such as agitation from anesthesia recovery. Skin from mice submitted to puncture and cell culture medium M199 was used as control. Skin from mice submitted to the bite of uninfected nymphs was sampled 24 h after the biting and used as a control for saliva deposition.

For molecular experiments that evaluated skin samples, mice were anesthetized, and an area of ~3 mm^2^ in the central region of the abdomen was delimited. After removal of the hair from this area, each mouse was exposed to bites of three *T*. *rangeli*-infected 5^th^ instar nymphs, starved for approximately 30 days. Each insect was allowed to remain with the mouthparts inserted into the host skin for approximately 10 sec, after which the process of probing was interrupted to prevent cannulation of blood vessels. The procedure was repeated for 1 min after which three other starved insects were used (n = 30 insects for each mice).

Mice used to evaluate the presence of *T*. *rangeli* in blood, lymph nodes and spleen were anesthetized, and an area in the central region of the abdomen was delimited. Each animal was exposed to three *T*. *rangeli*-infected 5^th^ instar nymphs, starved for approximately 30 days. Each insect was allowed to feed for 30 min, or until engorged.

Mice were analyzed for the presence of parasites in different tissues and organs, at different times after infection, henceforth referred to as hpi (hours post-infection) or dpi (days post-infection) as described elsewhere.

### Histological analysis

Skin samples were obtained through a microbiopsy of the delimited area using a dermatological punch with 5 mm^2^ diameter (Kolplast LTDA, São Paulo, Brazil) and all animals were euthanized immediately after this procedure. Skin samples were excised at 0 and 12 hpi, 1, 2, 4, 7 and 15 dpi (from three different mice for each evaluated time point) and kept frozen at -20°C until being further processed for histology techniques. Samples were then fixed in 4% paraformaldehyde diluted in phosphate buffer (0.1M, pH 7.2–7.4) for 24 h at 4°C, before being dehydrated and included in Paraplast (Sigma Aldrich Chemie, Deutschland, Germany). Sections of 7 μm were routinely stained with hematoxylin and eosin (HE) and then examined under an Axioplan 2 Zeiss microscope (Campbell, USA), equipped with HBO 100 W mercury lamp.

### DNA quantification analyses

#### DNA extraction

DNA extraction was performed using the Wizard Genomic DNA Purification Kit (Promega Corporation, Madison, USA), following manufacturer’s instructions, for all samples. Skin, mesenteric lymph nodes and spleens were homogenized in lysis solution with the aid of scissors, forceps and pestles. DNA extracts were quantified on a NanoDrop Spectrophotometer device (Thermo Scientific, Wilmington, USA) in order to determine concentration and quality assessment.

Skin samples were collected for different mice at the following time points (n = 4–10 for each evaluated time point): 30 min, 1–7 and 15 dpi. The whole area subjected to triatomine bite was excised by microbiopsy using a 3 mm^2^ diameter dermatological punch (Kolplast LTDA, São Paulo, Brazil). All animals were euthanized after this procedure and the samples were kept frozen at -20°C until further processing. Time points of 30 min, 2, 4, 7, 15 and 30 dpi were used for the analyses of blood, mesenteric lymph nodes and spleen samples (n = 5–12 for each evaluated time point). Blood (~1 ml) was collected by cardiac puncture and immediately transferred to a microtube containing 0.5M EDTA. Mesenteric lymph nodes and spleens were weighed and transferred to microtubes containing PBS. Samples were stored at -20°C until being further processed by DNA extraction.

#### Quantitative PCR (qPCR)

Reactions were performed with 1 μl of DNA extract containing 50 ng of DNA (except for extracts from blood samples which were less concentrated), 5 μl of Master SYBR Green PCR Master Mix (Applied Biosystems, Waltham, USA) and 0.5 μl (10 μM) of each primer, in a final volume of 10 μl. Experiments were conducted at the qReal-Time PCR Facility–RPT09D PDTIS/René Rachou Institute/FIOCRUZ MG in an ABIPRISM 7500 Sequence Detection System (Applied Biosystems, Waltham, USA). The following pair of primers was designed to amplify a 105 bp fragment of the *T*. *rangeli* annotated *KMP-11* gene: KMP84_F: GAAGTTCTTTGCGGACAAGC and KMP188_R: TTGAACTTGTCGGTGTGCTC. Parasite numbers estimation on different tissues was performed using corresponding standard curves, which were present in all qPCR plates. Standard curves consisted of a 7-point 5X dilution starting with an amount of DNA content equivalent to 10^5^ parasites prepared as follows: culture epimastigotes were added to a tube containing the same amounts of each tissue/organ used to quantify the samples from a uninfected mouse and subjected to DNA extraction as previously described. One microliter of this sample was used as the highest concentration of a standard curve. Tissue samples obtained from uninfected animals were used as a negative control to confirm parasite-specific amplification of primers. The following conditions were used for amplification: 95°C: 10 min, followed by 40 cycles of 95°C: 15 sec, 60°C: 1 min. An analysis of the dissociation curve (Tm) was done to confirm the specificity of the reaction. Only runs with efficiency of the curves between 90–110% and a R^2^ value around 0.99 were used for analyses.

#### RNA analyses

In addition to DNA analyses, RNA analyses were also performed to support our results showing accumulation of *T*. *rangeli* in lymph nodes and spleen of infected mice. RNA extraction, cDNA synthesis and qPCR were performed from lymph nodes and spleen samples and subjected to sequencing. TRIzol reagent (Invitrogen, Carlsbad, CA, US) was used for total RNA extraction according to the manufacturer’s instructions. RNA extracts were quantified using a NanoDrop Spectrophotometer, and treated with DNAse using RQ1 RNase-Free DNases (Promega, Fitchburg, WI, US) before being used (2 μg of total RNA) for reverse transcription using M-MLV Reverse Transcriptase (Promega, Fitchburg, WI, US). A new primer called KMP269 which matches the sequence GGGAACTGAGCGTTCTTCTG downstream of the pair of primers KMP84_F and KMP188_R was designed and used at 0.5 μl (10 μM) in a final volume of 12 μl to produce cDNA under the following conditions: 42°C for 60 min and 95°C for 5 min. For these experiments, mice were transcardially perfused with cold PBS immediately before the dissection of the organs. These cDNA were amplified by reactions performed with 1 μl of cDNA, 0.25 μl (10mM) of dNTPs, 0.5 μl (10μM) of each primer (KMP84_F and KMP188_R) and 1U of GoTaq polymerase (Promega, Fitchburg, WI, US) in a final volume of 12.5 μl. The following conditions were used for amplification: 35 cycles of 94°C: 45 sec, 60°C: 45 sec and 72°C: 1 min. PCR products were visualized in 2% agarose gels, excised and purified using the Wizard SV Gel and PCR Clean-Up System Kit (Promega, Fitchburg, WI, US) before being sequenced with both primers using an ABI Prism BigDye V 3.1 Terminator Cycle Sequencing kit and an ABI 3730 DNA sequencing system (Life Technologies, Carlsbad, CA, US).

### Imaging flow cytometry

Splenocytes were collected from infected and non-infected Swiss mice at 3, 10 and 30 days after infection. Briefly, each animal spleen was macerated with cell strainer and washed with RPMI 1640 supplemented with 1.6% L-glutamine. Erythrocytes were lysed using 20 mL of FACS Lysing Solution (BD Biosciences, USA) and washed with 20 mL of PBS (phosphate-buffered saline containing 0.01% sodium azide). Following, 1x10^7^ cells/mL were permeabilized in saponin buffer (0.5%) (Sigma-Aldrich, USA) for 10 minutes, stained with mouse anti-*T*. *cruzi* monoclonal antibody that recognizes the paraflagellar rod protein of the parasite [25] (kindly provided by Prof. Maria Julia Manso Alves), diluted 1:100 and incubated for 30 minutes at 4°C. After incubation, cells were washed with 3 mL of saponin buffer and stained with anti-mouse secondary antibody conjugated with Alexa Fluor 488 (AF488, BD Pharmingen, USA) diluted 1:500. Finally, cells were incubated in the dark for 30 minutes at 4°C and washed with PBS prior to imaging flow cytometry. *T*. *rangeli* parasites were stained in the same conditions as splenocytes. A total of 50,000 events/sample were run in an ImageStream Mark II Amnis flow cytometer and analyzed using Ideas software v.6.0 (Amnis, Luminex, USA).

### Lymph node and spleen weight analyses

To evaluate whether the weight of the mesenteric lymph nodes and spleen was affected by the parasite infection, five mice from time periods 30 min, 2, 4, 7 and 15 d had these organs weighed after dissection. Organs from uninfected control animals from the same batch and kept at same conditions were also processed at the same time periods. To remove any effect of different animal sizes, the weights of the organs were normalized by each animal full body weight.

### Statistical analysis

The amounts of parasite DNA found in different days of each tissue/organ were compared using the Kruskal-Wallis non-parametric test. Pairwise comparisons were performed by means of Dunn’s test. The proportional weights of lymph nodes were analyzed by two-factor ANOVA using infection and time as independent variables. The proportional weights of spleens were analyzed by general linear models (GLM) to identify the variables affecting the parameter weight, followed by the Kruskal-Wallis non-parametric test to compare the weight of spleen over time. Pairwise comparisons were performed by means of Dunn’s test.

## Results

### The presence of *T*. *rangeli* causes changes in the skin of the mammalian host

**Fig 1:**
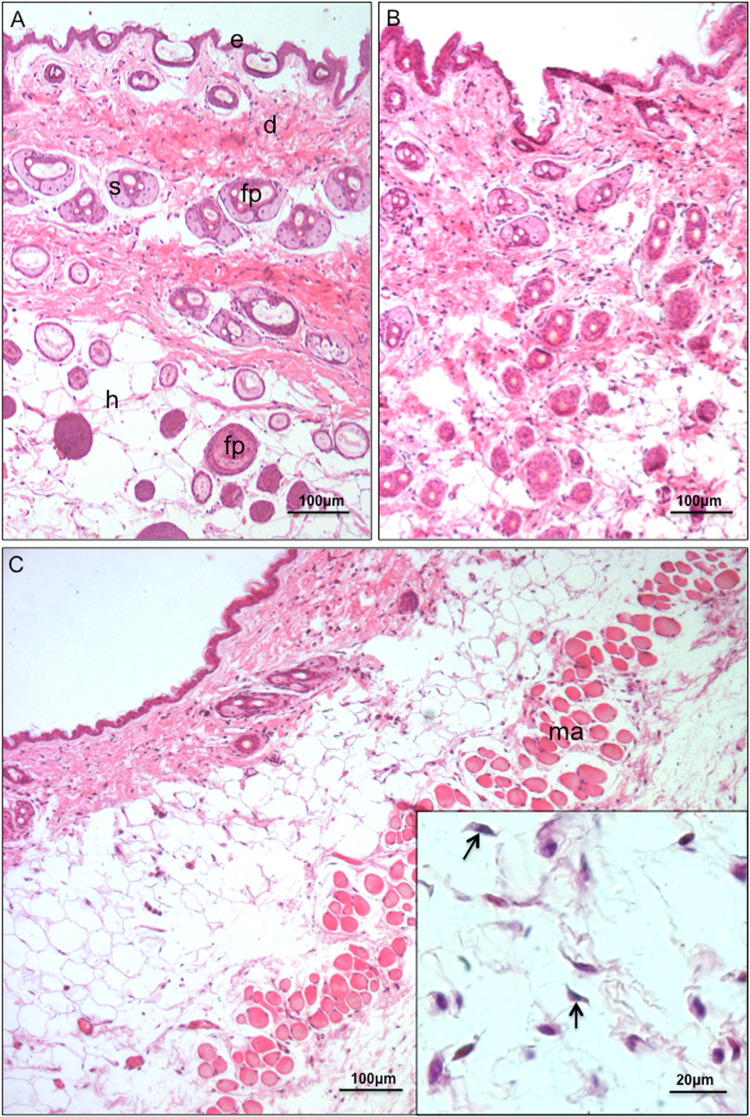
*Trypanosoma rangeli* can be observed in the skin of infected mice immediately after infection. HE-stained photomicrographs of histological sections of mice skin immediately after exposure (0 h). In both, control skins (A) and control punctured skins immediately after puncture and addition of M199 medium (B), typical layers (e = epidermis, d = dermis, h = hypodermis) and appendages (fp = hair follicles; s = sebaceous glands) are observed. No inflammatory infiltrates are observed in any of the controls. In the skin of animals that were exposed to trypomastigotes immediately after the procedure (C), numerous parasites concentrated in the hypodermis region (insert, arrows) can be observed. Inflammatory infiltrates are not observed at this period. Part of the abdominal musculature (ma) can also be observed in the section.

**Fig 2:**
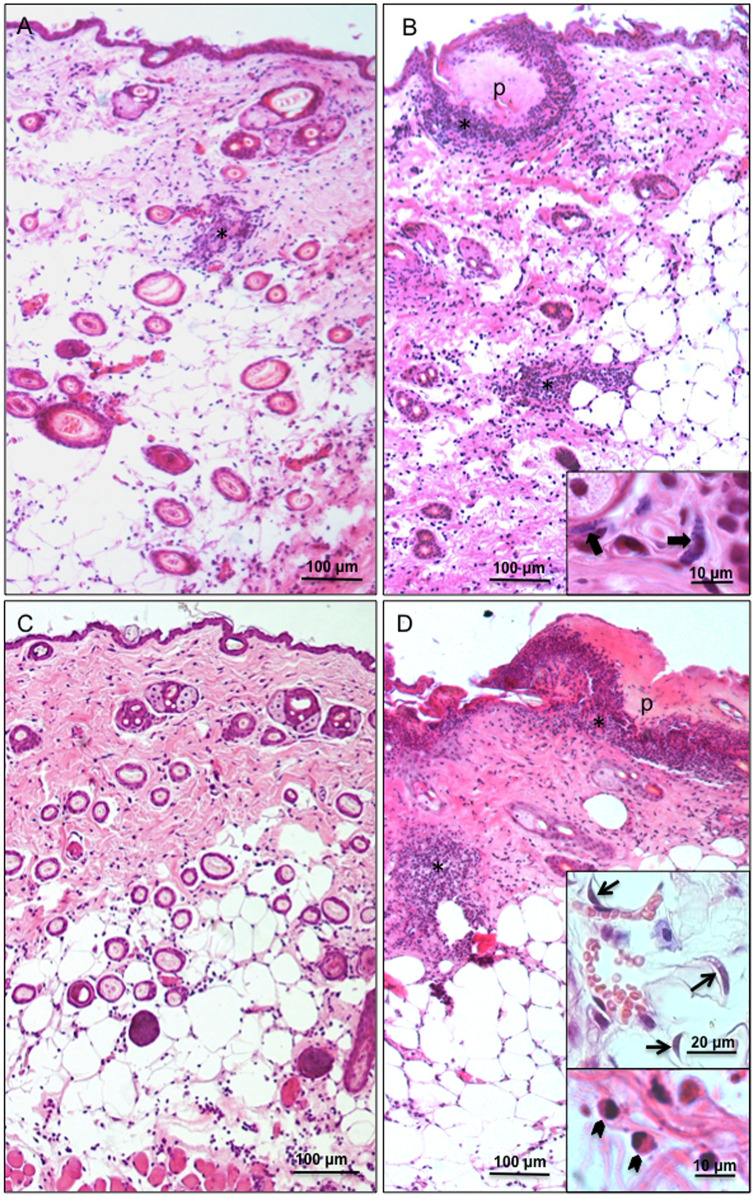
*Trypanosoma rangeli* presence starts inflammatory responses as early as 24 h post-infection. Photomicrographs of histological sections stained with HE of mice skin at 12 h and 1 day post-infection. Twelve hours after puncture, in samples exposed to M199 medium there is diffuse discrete inflammation and rare inflammatory foci (asterisk) (A). Only mild diffuse inflammation is observed after 1 d of M199 medium exposure (C). Important inflammatory infiltrates, both diffuse and focal (asterisks), are observed in the skin of animals exposed to trypomastigotes after 12 h and 1dpi (B and D, respectively), being remarkable the presence of numerous mast cells (insert in B, broad arrow) and eosinophils (insert in D, arrowhead), in addition to typical mononuclear cells. Trypomastigotes were still visualized in the hypodermis 1dpi (insert in D, arrows). In both times of exposure, intense inflammatory focus at the puncture site is also observed (p).

**Fig 3:**
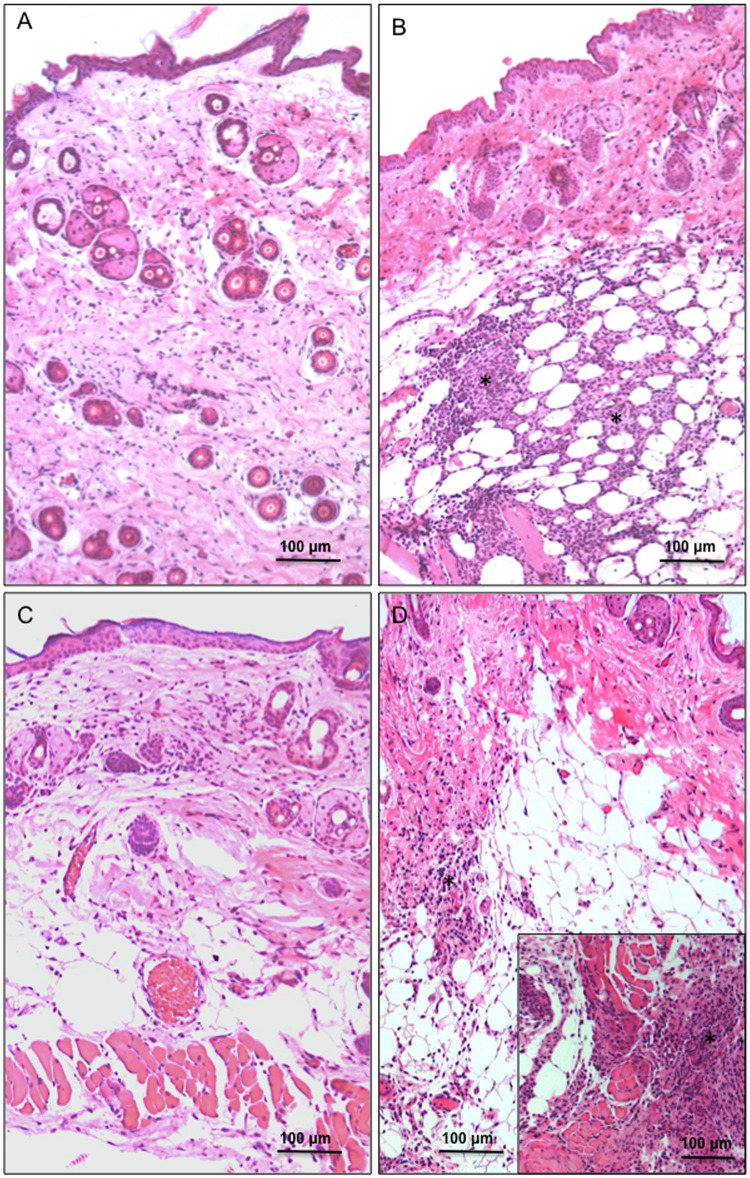
*Trypanosoma rangeli* promotes robust inflammatory response in the skin of infected mice. Photomicrographs of histological sections stained with HE of mice skin at 2 and 4 days post-infection. No inflammatory infiltrates are observed in the skin of animals exposed to M199 medium at two (A) and four (C) dpi. In the skin of animals with 2 dpi to trypomastigotes (B), although no more parasites are observed, the inflammatory process (asterisks) persists, especially in the region of hypodermis and more intense than that seen in previous times of exposure. At 4 dpi (D), the inflammatory process intensifies further, with large foci of inflammatory cells in the hypodermis and dermis (asterisks in D and insert).

In depth analyses of the alterations caused by *T*. *rangeli* infections on mammalian skin are yet to be characterized and to shed light into this subject we evaluated the changes that take place in injured skin of mice upon exposition of *T*. *rangeli* metacyclic trypomastigotes. Application of salivary gland extracts of *T*. *rangeli*-infected triatomines to the purposely injured skin from abdominal areas of mice were carried out to evaluate its effects on skin integrity. Histological sections obtained from control animals in which no injury or infection procedure were carried out showed an expected morphology of typically healthy skin and absence of inflammatory processes (Fig 1A). Analyses of histological sections of punctured uninfected controls immediately after the procedure (0 h) also showed typical skin structures and absence of inflammatory infiltrates (Fig 1B). Twelve hours after the puncturing procedure some inflammatory foci could be identified on the dermis of these samples, mainly near the punctured area (Fig 2A). A reduction of these inflammatory infiltrates was already observed after 24 h of M199 exposition (Fig 2C). Similar results were observed for samples taken from mice that had been subjected to the bite of uninfected nymphs (S1 Fig). Evidence of tissue repair and a gradual decrease in inflammatory infiltrate were consistently observed in samples from control uninfected animals on subsequent days (Fig 3A and 3C, S2A and S2C Fig).

Histology results were able to clearly identify metacyclic trypomastigotes in dermal and hypodermal areas of the parasite-exposed skin immediately after the procedure, henceforth referred to as 0 h (Fig 1C). No inflammatory infiltrates were observed at this time (Fig 1C). After 12 h of infection, parasite-induced inflammatory infiltrates were prominently observed on the dermis and hypodermis near/adjacent to the needle insertion site (Fig 2B). A remarkable number of mast cells and eosinophils were found in these inflammatory infiltrates, as well as typical mononuclear cells (Fig 2B). Trypomastigotes were likewise observed at the wounded area of samples collected at 1 dpi although in a lower density than that found at 0 h (Fig 2D). The inflammatory profile was similar to that observed for 12 hpi, with presence of mononuclear cells, eosinophils and mast cells (Fig 2D; a more detailed picture of the cells present in the inflammatory infiltrates is shown in S3 Fig). No parasites could be visualized in sections from skin collected at time points of 2 dpi. Noteworthy, inflammatory processes seem to have spread to wider areas of the dermis and hypodermis of animals from 2, 4 and 7 dpi when compared to skin sections from 1 dpi samples (Fig 3B and 3D and S2B Fig). Inflammatory infiltrates were no longer observed in histological sections examined from samples collected at 15 dpi (S2D Fig).

### *T*. *rangeli* DNA can be detected at its inoculation site on murine skin for a week after infection

**Fig 4:**
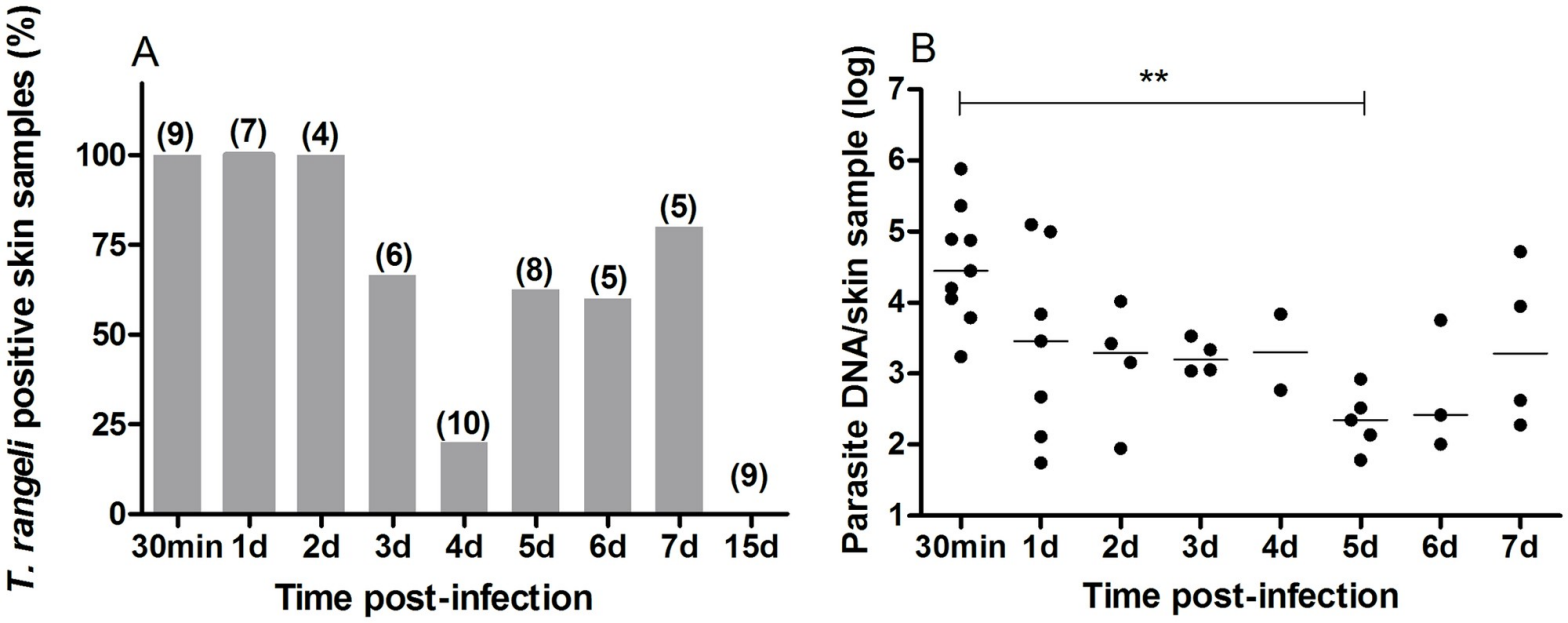
*Trypanosoma rangeli* can be detected on mouse skin up to seven days post-infection. (A) Percentage of skin samples showing positive amplification of *T*. *rangeli* DNA by qPCR for each day. The values in parentheses indicate the number of samples analyzed for each time point. (B) Quantification by qPCR of *T*. *rangeli* DNA in infected mice skin samples. Each dot represents the estimated number of *T*. *rangeli/*sample. Horizontal lines represent the median of values found for each time period. The Y-axis shows values on a logarithmic scale (Kruskal-Wallis, n.s.).

Aiming to evaluate the persistence of *T*. *rangeli* at parasite entrance gate on the skin of infected mice, we used qPCR to identify the presence of its DNA at different time points after triatomine biting. Interestingly, our results suggest that parasites can persist on murine skin for several days once they are injected during vector´s vessel-probing phase. Amplification of *T*. *rangeli*-specific DNA, which corresponds to a 105-bp segment of its *KMP11* gene, identified the presence of parasite DNA from day 1 up to day 7 pi. Percentage of positive samples varied considerably over this period ranging from 20% at day 4 to 100% at 30 min and days 1 and 2 pi (Fig 4A). At least four animals were screened for each experimental time period, and qPCR analyses of 9 skin fragments from different samples collected from mice at 15 dpi failed to detect parasite DNA at this time point. Estimation of parasite numbers in the positive samples showed differences in the amount of parasites over time (Fig 4B; Kruskal-Wallis, p = 0.02). A significant decrease in DNA content was observed from the initial assessment (30 min after infection) to the 5^th^ dpi (Dunn’s Multiple Comparison, p<0.05).

### *T*. *rangeli* DNA can be detected in circulating blood and lymphoid tissues of infected animals for up to 30 days post-infection

**Fig 5:**
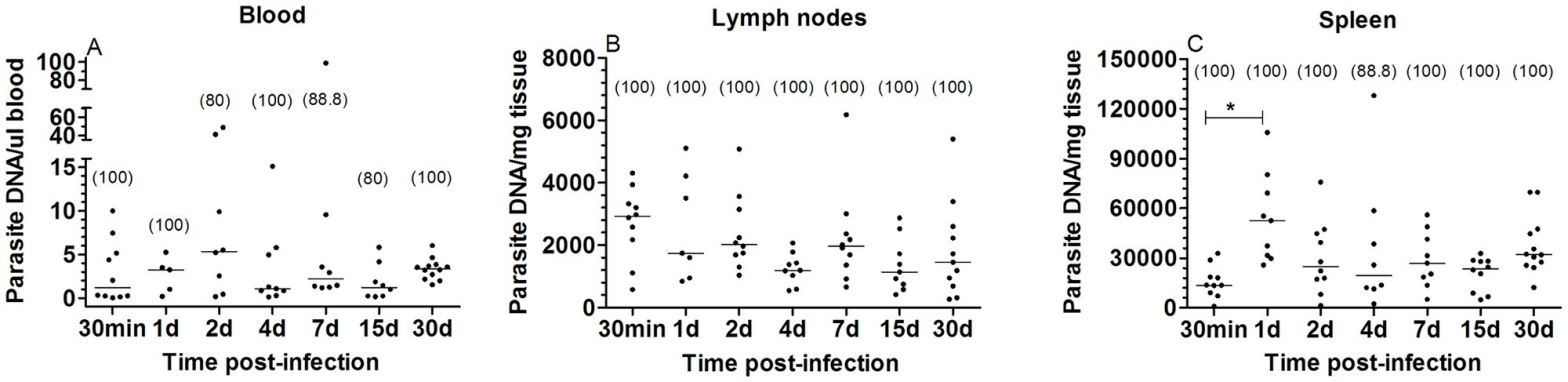
*Trypanosoma rangeli* can be detected in different mouse tissues up to 30 days post-infection. Quantification by qPCR of *T*. *rangeli* DNA in blood (A), mesenteric lymph nodes (B) and spleen (C) from infected mice samples. The values in parentheses indicate the percentage of samples showing positive amplification for each time point. Each dot represents the estimated number of *T*. *rangeli/*μl of blood or μg of tissue. Horizontal lines represent the median of values found for each time period.

After confirming that *T*. *rangeli* is not able to establish a multiplicative population in the inoculation site, we searched for possible tissue/organs targeted by the parasite after it leaves the skin. Therefore, we evaluated whether parasites replicate in the blood, also in mesenteric lymph nodes, which might receive parasites captured by the lymphatic system during the probing phase, and spleen, where blood-circulating parasites could be retained. We analyzed infected mice at different time points from 30 min up to 30 dpi and our results demonstrated a high prevalence, over 80%, of *T*. *rangeli* DNA in all blood samples analyzed (Fig 5A). Higher variations on the amount of parasite DNA were observed during the first week of infection (ranging from 0.03 to 98.9 par/μl), in comparison to days 15 and 30 pi when the amount of *T*. *rangeli* DNA was more homogenous (ranging from 0.24 to 6.03 par/μl). Nevertheless, no significant differences were observed in the amount of parasite DNA detected over the evaluated period (Fig 5A; Kruskal-Wallis, n.s.). Our analyses were able to identify *T*. *rangeli* DNA in the lymph nodes of all infected animals, and 98.5% of all spleen samples, being the only exception a single sample from a 4 dpi infected mouse. In addition of being detected in all lymph nodes, at different evaluated time points, the amount of *T*. *rangeli* DNA remained fairly unchanged over the 30 days period (Fig 5B; Kruskal-Wallis, n.s.). As for the amount of parasite DNA in spleen samples, there was a variation over the evaluated period (Fig 5C; Kruskal-Wallis, p = 0.005), in which the amount of parasites showed an increase from 30 min to day 1 pi (Fig 5C; Dunn’s Multiple Comparison, p<0.05). Interestingly, we were able to detect *T*. *rangeli* DNA in lymph nodes and spleen of all 5 animals from which qPCR was unable to detect DNA in their blood samples.

**Fig 6:**
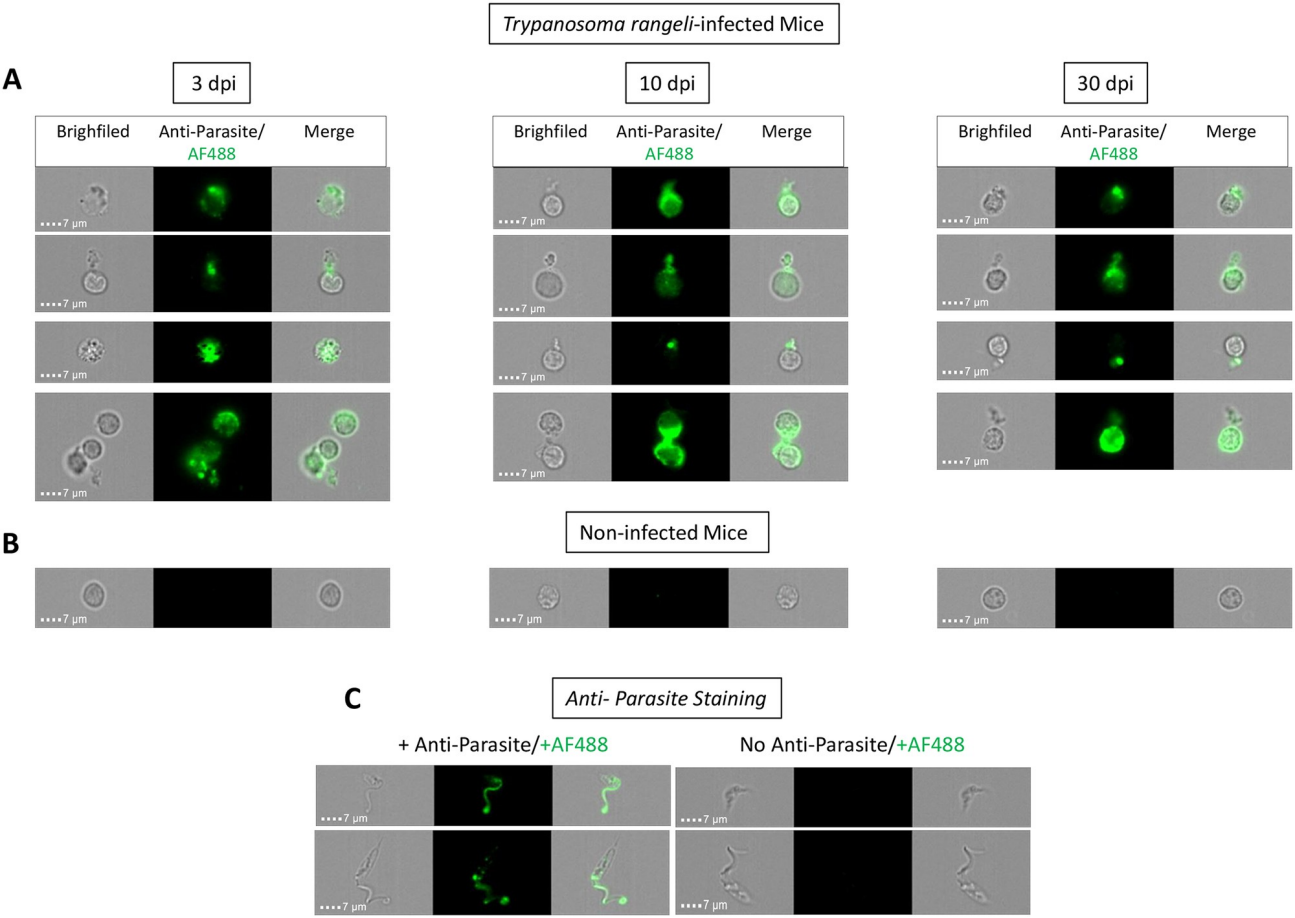
Live *Trypanosoma rangeli* parasites can be detected in spleen up to 30 days post-infection. Imaging flow cytometry was used to identify *Trypanosoma rangeli* forms in splenic cell suspension of infected (A) and non-infected mice (B) in distinct days of infection (3, 10 or 30 dpi). Detailed morphometric cellular/parasite analyses were performed employing brightfield channel, green fluorescence channel (mouse anti-*T*. *cruzi* antibody + anti-mouse-Alexa Fluor 488) and merged channels. (D) Additional analysis to confirm the monoclonal antibody specificity was also performed using cultivated *T*. *rangeli* epimastigotes. In all cases, representative image galleries of cells were generated in 600 x objectives/bar = 7mm.

To confirm the presence of live parasites evidenced by *T*. *rangeli* DNA, we decided to use two different strategies. First, we sequenced cDNA samples obtained from RNA extracts of lymph nodes and spleens from infected mice. BLAST analyses confirmed the presence of *T*. *rangeli*-specific sequences in these RNA extracts (accession number MN864064 and MN864065, respectively). In addition, we analyzed suspensions of splenic cells by imaging flow cytometry and searched for parasites in infected animals. Animals infected for 3, 10 and 30 days were used in these analyses and our results identified the presence of parasites in splenic suspension at all time points (Fig 6A). Moreover, the parasite seems to be interacting with splenic immune cells in all evaluated time points, as shown in representative images (Fig 6A). In the images, it is possible to visualize the parasites in the brightfield channel and after anti-*T*. *cruzi* antibody staining. To confirm the specificity of the parasite antibody, cultivated epimastigotes of *T*. *rangeli* were stained with the same reagent (Fig 6C).

**Fig 7:**
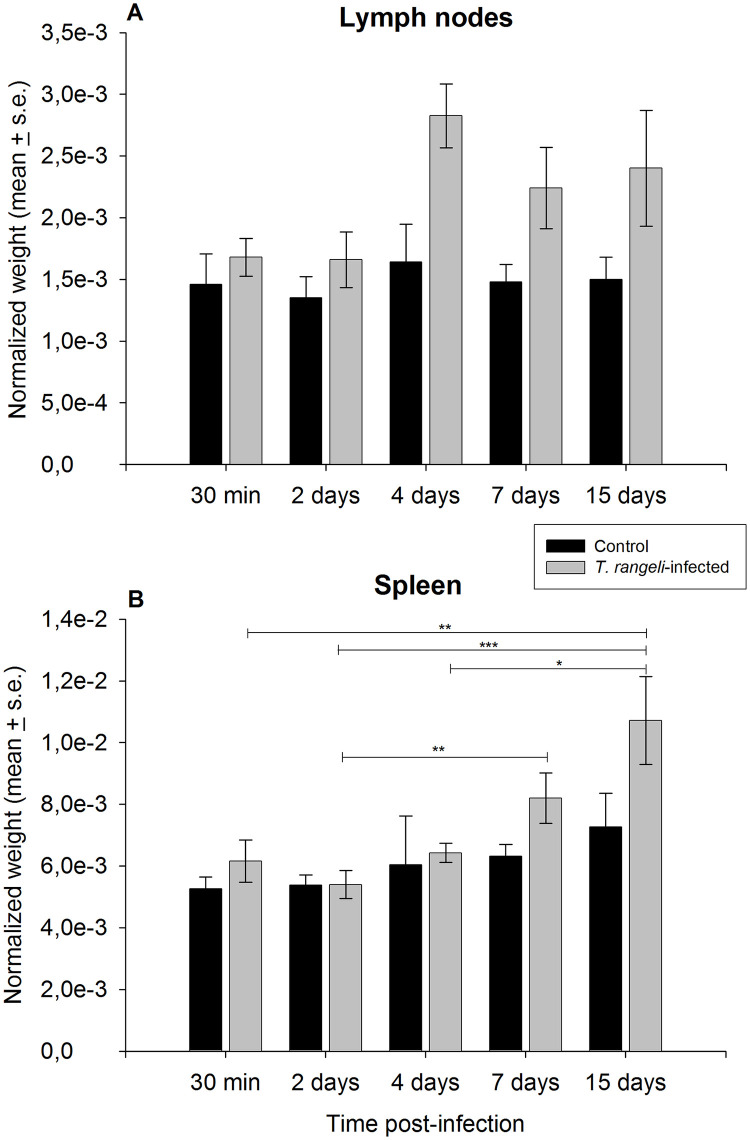
*Trypanosoma rangeli* infection increases lymph nodes and spleen size. Normalized weight of mesenteric lymph nodes (A) and spleen (B) from mice infected or not by *T*. *rangeli* in different days post-infection. Data represents the mean ± s.e. of 4–5 animals.

In addition to the high amount of parasites, clear differences in organ size were observed upon dissection of lymphoid tissues from *T*. *rangeli*-infected and control animals. Therefore, the weight of lymph nodes and spleens was analyzed in animals from time points of 30 min, 2, 4, 7 and 15 dpi. An increase in lymph node mass was observed in infected mice (Fig 7A, ANOVA; infection p = 0.0003). Spleen weight also showed an increase in the infected mice in comparison with control animals (Fig 7B). Our analyses demonstrated that, differently from observed for lymph nodes, spleen weight was affected by either experimental time, i.e., control and infected mice showed spleen mass growth in subsequent time points, as well as time of infection, for which a parasite-related increase was observed after the 7 dpi time point (Fig 7B, GLM; infection p = 0.01, days p = 0.001; Dunn’s Multiple Comparison for infected group, 7 *vs* 2d p = 0.008, 15 *vs* 30min p = 0.009, 15 *vs* 2d p = 0.0004, 15d *vs* 4d p = 0.01).

## Discussion

Understanding of many aspects of the biology of interactions between *T*. *rangeli* and its vertebrate host has been challenging scientists for several decades, mainly due to the fact that this parasite promotes an asymptomatic infection. Our present work used *in vivo* analyses of naturally infected mice to track down parasites from their entry site to putative target development organs, and we were able to show, for the first time, that *T*. *rangeli* can be detected in the skin (for up to a week), blood, mesenteric lymph nodes and spleen of a mammalian host for up to 30 days.

Protozoan parasites undertake different strategies to evade vertebrate defense mechanisms to find proper conditions for the development of their replicative forms in order to complete their life cycle. Presence of parasite replicative niches in mammalian skin has been reported for *Trypanosoma brucei* and *Plasmodium berghei* in association with dermal adipocytes and hair follicles, respectively [26–28]. Therefore, we set out to investigate whether similar strategies could be related to *T*. *rangeli* survival in mice, and our histology and qPCR data from *T*. *rangeli* tracking in mice skin showed that although we were able to detect the presence of parasites at its inoculation site on the skin in 100% of infected samples until the 3^rd^ dpi, a clear decrease of parasitemia and possible clearance of skin infection was observed at the end of a 15 days period of analyses. qPCR analyses showed that, in general, parasite numbers in the skin were highly variable and decreased over time reaching significance on day 5 pi. No positive samples were found on day 15 pi, indicating that the parasite was eliminated from the skin, or was present at amounts too low to be detected by the strategies used in the present work. Noteworthy, it is important to point out that our data includes independent experiments performed over different times with different batches of mice and parasites. Interestingly we were only able to visualize parasites in histological sections of samples collected during the 1^st^ dpi in which only extracellular parasites were observed in the dermis and hypodermis. Detection of *T*. *rangeli* was confirmed by qPCR during the first 7 days after infection and further evidence of a focal skin parasite infection was supported by pronounced inflammatory infiltrates formation during this first week. Large numbers of mast cells and eosinophils at the delivery site, usually observed also in other protozoan infections [29–31], are probably related to the parasites clearance from skin. It is worth to point out that recruitment of immune cells to infected skin occurs not only in response to pathogen challenge, but also due to physical injury and deposition of saliva components from haematophagous invertebrate vectors during blood feeding [32–35]. This diverse group uses different strategies to extract blood from their vertebrate hosts. While some species are telmophagic and take blood from a hemorrhagic pool formed on the dermis, others are solenophagic and withdraw their meal directly from vessels [18,36,37]. In common, all hematophagous arthropods secret a saliva composed by a cocktail of proteins, including anticoagulants, anti-platelet aggregation, anesthetics, vasodilators and immunomodulators, among others, to delay blood haemostasis and increase their chances of feeding (reviewed by [38,39]). In the case of triatomine bugs, the deposition of saliva starts just after the introduction of the mouthparts in the host skin and continues uninterruptedly throughout the probing and ingestion phase [19]. For this reason, we cannot neglect the contribution of triatomine saliva to the recruitment of immune cells, although there are clear differences in the cellular profile found on parasite-infected and uninfected injured skins. *T*. *rangeli* presence seems to be related to monocytes and lymphocytes accumulation as well as a persistent diffuse inflammation after the second day post-infection, which is not observed after 24 h in control animals exposed to culture medium or bites of uninfected insects (S1 Fig). Therefore, it seems reasonable to suggest that the largest proportion of immune cells present at the infected area is, in fact, due to the immune response triggered by *T*. *rangeli*. Additionally it has been demonstrated that triatomine infection with *T*. *rangeli* promotes a decrease in approximately 50% of the expression of stocked proteins in the salivary glands of *R*. *prolixus* [24]. Such decrease might have reduced cell recruitment effect as a result of saliva deposition. The absence of parasite DNA detection at the 15^th^ dpi in association with clearance of skin inflammation at the same period also suggests that *T*. *rangeli* is eliminated from its inoculation site, which, according to our results does not seem to behave as a parasite multiplication niche.

Blood is the place where *T*. *rangeli* must circulate to guarantee its transmission to the triatomine host. Low numbers of parasites were consistently observed in blood from infected mice, in agreement with data from fresh examinations [8–10]. The role of circulating blood as a dissemination route instead of an established infection site is corroborated by the findings that for some of the samples evaluated in this study we were not able to detect parasite DNA in circulating blood even though lymphoid organs of the same animal were positively infected. Nevertheless, a small number of circulating parasites does not seem to affect the rates of *T*. *rangeli* transmission from infected mice to triatomines which are close to 90% [15].

Interestingly, the presence of parasites in the mesenteric lymph nodes as soon as 30 min after the end of the feeding indicates that part of the parasites which are released with the saliva in the dermis while the insect is searching for a blood vessel is rapidly transported to the lymphatic system. Whether the parasite is taken up by or invades some circulating cells, is drained extracellularly by the lymph, or yet, invades actively the lymphatic vessels, remains to be determined. Lack of variation in parasite numbers found over 30 days in the lymph nodes suggests that this tissue may act as a maintenance site for *T*. *rangeli*. The observation that no parasites or inflammation are present in the skin 15 d after the infection corroborates this hypothesis. Despite its main function as an immunity related organ, lymph nodes have been reported to maintain transitory and stable populations of live pathogens such as the *Yersina pestis* [40,41], *Theileria parva* [42], *T*. *brucei* [26] and *Leishmania sp*. [43]. *Plasmodium* parasites have also been showed to actively invade lymphatic vessels [44]. In a model of *P*. *berghei*-infected mice, most sporozoites were degraded during the first hours after invading lymph nodes, but some can mature into exoerythrocytic forms and partially develop in this organ. Interestingly, the same study showed a 20-fold higher amount of parasites in lymph nodes when mice infections were performed naturally through the bite of an infected mosquito together with saliva as opposed to injection of sporozoites extracted from salivary glands [44].

From all analyzed tissues, the highest concentrations of *T*. *rangeli* DNA were found in the spleen of infected mice in which the average number of detected parasites was around 10 and 10^3^ times higher than those observed in lymph nodes and blood, respectively. The peak of *T*. *rangeli* accumulation observed at 24 hpi might be in fact associated with an immunity-related retention of the initial vector-inoculated parasite circulating in blood. After this, parasite numbers in spleen reached a steady state from 2^nd^ to the 30^th^ dpi. Therefore, these results and the identification of intracellular as well as free forms of *T*. *rangeli* in the spleen at 3, 10 and 30 dpi raise a hypothesis that the organ might provide a possible survival and multiplication site for the parasite. In agreement with our results, the spleen has also been reported as a multiplication site for other trypanosomatids, such as *Leishmania* [45,46], and *T*. *cruzi* [47]. In the case of *Leishmania chagasi*, per example, experimental infections in hamsters showed histological changes in the spleen, which were accompanied by a progressive increase in parasite amounts [48]. More recently, luciferase imaging associated with RT-qPCR has shown that *Leishmania donovani* can remain in mice spleen for over six months, with an increase in parasite burden in later periods when compared with the first month of infection [49]. For *T. cruzi*, parasites of diverse strains have been found in the spleen. Mice infected with Tulahuen strain and evaluated by qPCR showed mean levels of ~10,000 parasitic DNA equivalent parasites per 50 ng of total DNA on 21 dpi. The numbers were reduced by the 74 dpi but restored after the administration of the immunosuppressive cyclophosphamide [47]. Mice orally infected with Dm28c strain and evaluated by luciferase imaging and qPCR also showed parasites in spleen in both 7 and 21 dpi [50]. In addition, a next-generation metabarcoding characterization of the full complement of discrete typing units (DTUs) of rodents naturally infected with *T*. *cruzi* showed a diversity of haplotypes present in the spleen of *Peromyscus gossypinus* mice collected in a rural area of New Orleans metropolitan region (Louisiana, USA) [51].

It is interesting to note that on the 4^th^ dpi *T*. *rangeli*-positivity rate in the skin reached the lowest, at 20% and returned to higher values on subsequent days. Similarly, average parasite numbers in blood, lymph nodes and spleen were also lower on the day 4 pi at the same time period in which the increase in lymph nodes weight was observed. Although yet speculative, these observations could altogether suggest that some process of parasite change or host response might be happening around the middle of the first week of infection and future studies are necessary to clarify this point of the infection.

In conclusion, using naturally infected mice we have detected for the first time stable populations of *T*. *rangeli* in secondary lymphoid tissues, suggesting that the parasites that are released in the host´s skin and blood circulation can establish stable infections in the mammalian host. It is important to highlight that in the present study we used a methodology of infection where the parasites entered in the vertebrate host by a natural way of delivery, in addition of using a *T*. *rangeli* strain that was frequently passaged through mammal and insect hosts to guarantee its infectivity. This, together with the use of more sensitive techniques such as the qPCR and imaging flow cytometry, may explain the unsuccessfully attempts of previous studies in detecting the parasite in mammals [9,14,16,17]. Further perspectives of work include (i) assessment of the route used by *T*. *rangeli* to reach and colonize secondary lymphoid tissues; (ii) analyses of parasite-cell interactions and identification of the host cells invaded by the replicative forms of parasite; (iii) investigation of other tissue/organs in which the parasite may develop. Our new exciting results showing accumulation of *T*. *rangeli* on lymph nodes and spleen shed some light on the parasite´s development cycle in the vertebrate host. Therefore, investigating where and how *T*. *rangeli* replicates within mammalian hosts will unravel the mechanisms related to the balance between parasite dissemination and host immunological responses.

## Supporting information

S1 Fig Photomicrographs of histological sections stained with HE of mice skin submitted to bites from uninfected insects 1 day post-infection. In A, B and C, punctual inflammatory foci (asterisks) are observed in the hypodermis. It is possible to observe the presence of mononuclear cells in the infiltrates (B).
(TIF)

S2 Fig Photomicrographs of histological sections stained with HE of mice skin at 7 and 15 days post-infection. No inflammatory infiltrates are identified in the skin of animals exposed to M199 medium, both at 7 (A) and 15 (C) dpi. In the skin of animals exposed to trypomastigotes there is persistence of intense inflammation (asterisk) 7dpi (B). Fifteen dpi (D), no major inflammatory processes are observed.
(TIF)

S3 Fig Photomicrographs of histological sections stained with HE of mice exposed to *T*. *rangeli* trypomastigotes showing different cells found in inflammatory infiltrates. Monocytes/lymphocytes (narrow arrows), neutrophils (red arrowhead) and eosinophils (wild arrows) can be identified in samples exposed to parasites for 7 days.
(TIF)
