# Supplementary figures and images for: New features on the survival of human-infective Trypanosoma rangeli in a murine model: Parasite accumulation is observed in lymphoid organs
Source: PLoS Negl Trop Dis. 2020 Dec 28;14(12):e0009015. doi: 10.1371/journal.pntd.0009015 (PMC7793305; doi:10.1371/journal.pntd.0009015)

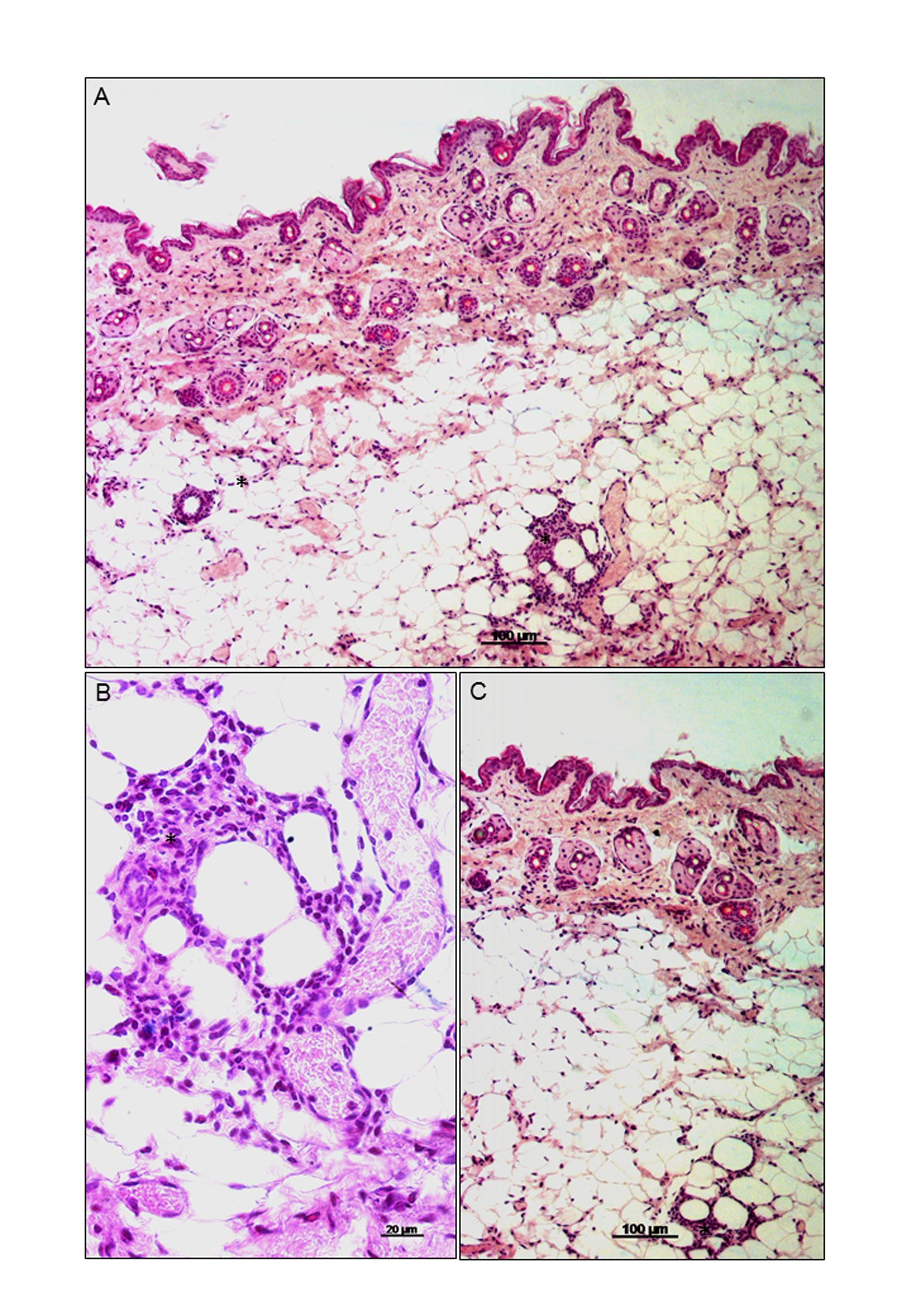

Supplement: S1 Fig — In A, B and C, punctual inflammatory foci (asterisks) are observed in the hypodermis. It is possible to observe the presence of mononuclear cells in the infiltrates (B). (TIF) [file pntd.0009015.s001.tif]

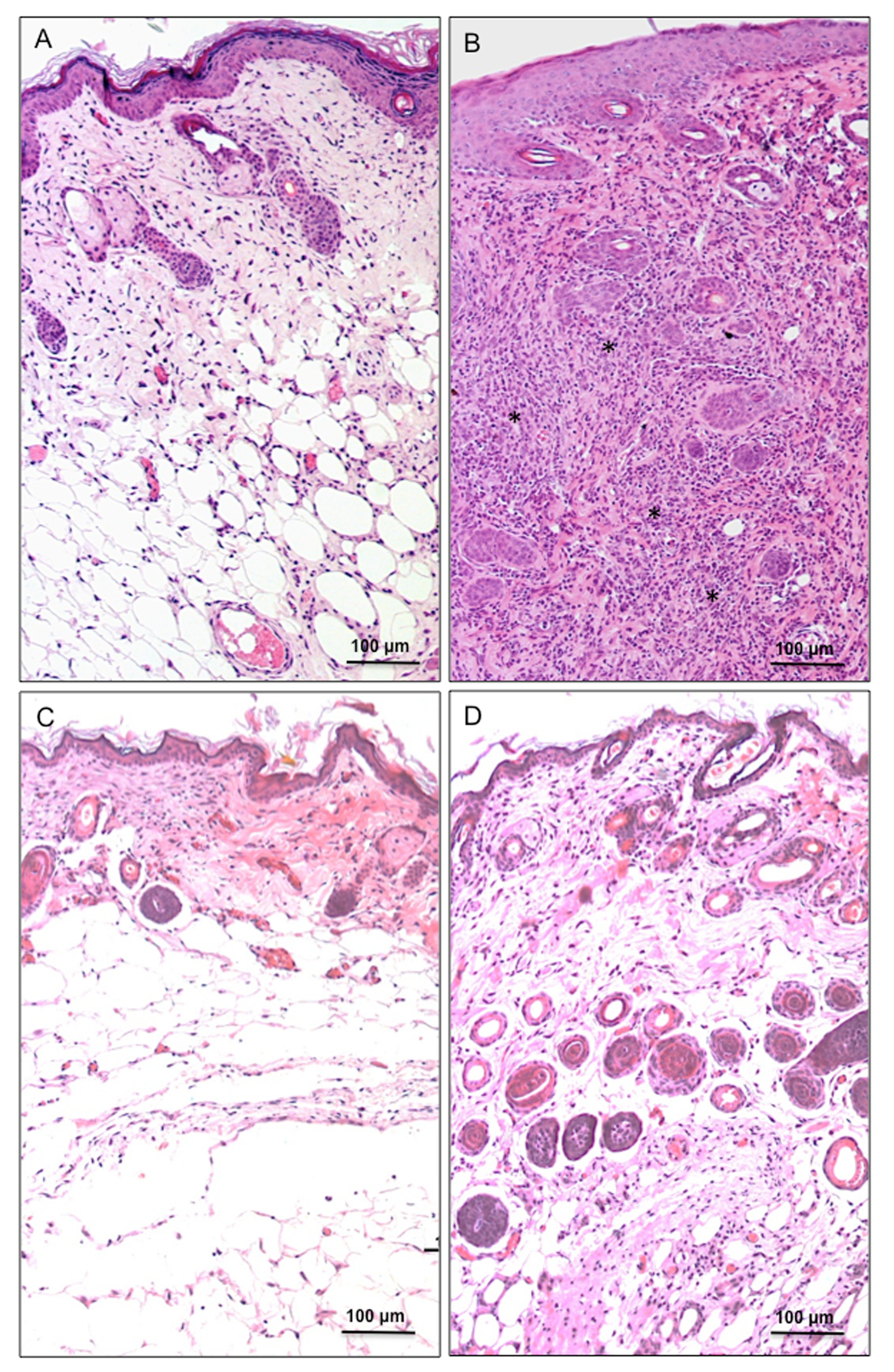

Supplement: S2 Fig — No inflammatory infiltrates are identified in the skin of animals exposed to M199 medium, both at 7 (A) and 15 (C) dpi. In the skin of animals exposed to trypomastigotes there is persistence of intense inflammation (asterisk) 7dpi (B). Fifteen dpi (D), no major inflammatory processes are observed. (TIF) [file pntd.0009015.s002.tif]

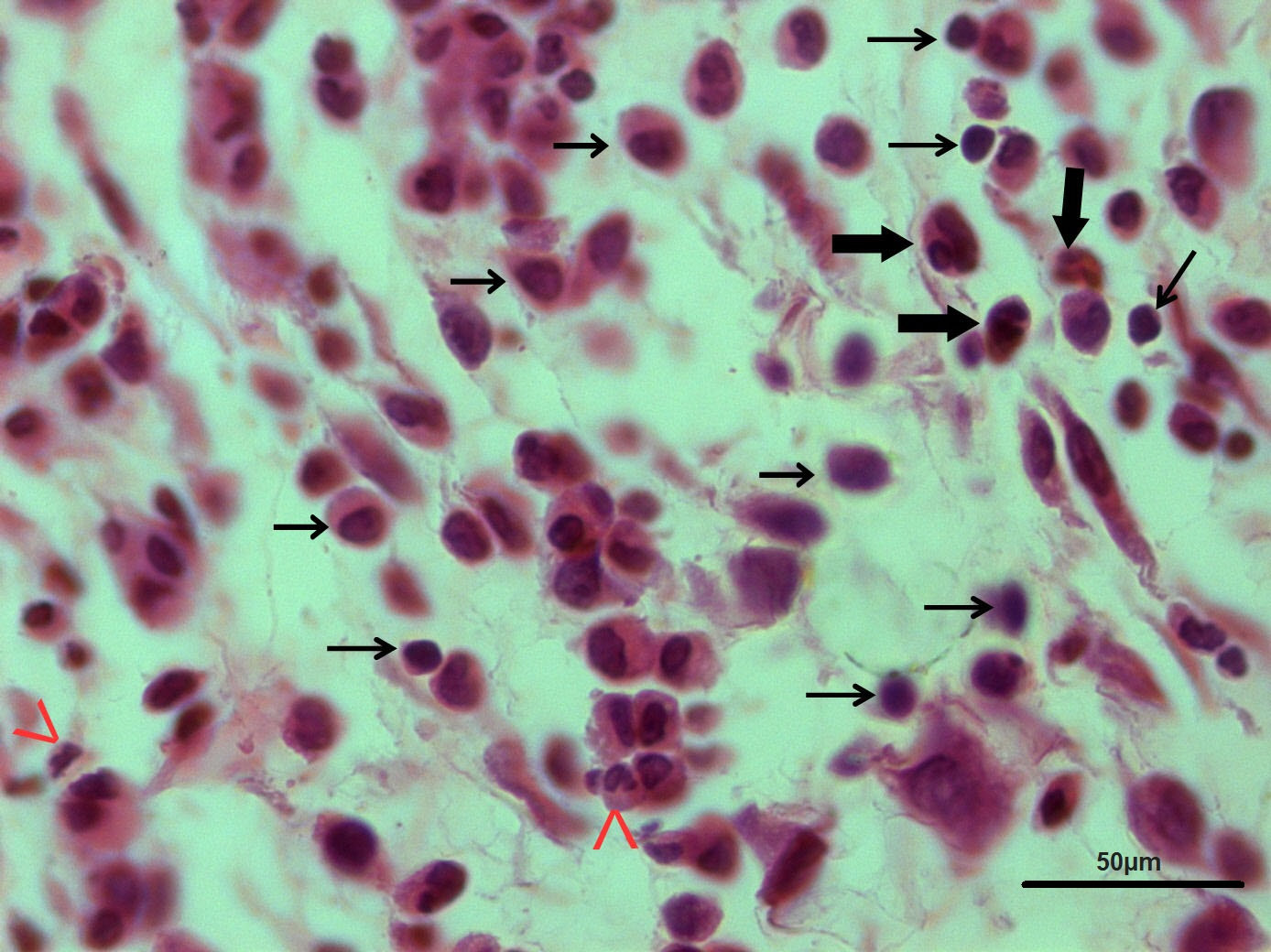

Supplement: S3 Fig — Monocytes/lymphocytes (narrow arrows), neutrophils (red arrowhead) and eosinophils (wild arrows) can be identified in samples exposed to parasites for 7 days. (TIF) [file pntd.0009015.s003.tif]
